# The role of TGF-β in the electrotactic reaction of mouse 3T3 fibroblasts *in vitro*

**DOI:** 10.3389/abp.2024.12993

**Published:** 2024-06-25

**Authors:** Patrycja Ciesielska, Slawomir Lasota, Sylwia Bobis-Wozowicz, Zbigniew Madeja

**Affiliations:** Department of Cell Biology, Faculty of Biochemistry, Biophysics and Biotechnology, Jagiellonian University, Kraków, Poland

**Keywords:** electric field, electrotaxis, cell migration, TGFβ signaling, 3T3 fibroblasts

## Abstract

Endogenous electric fields (EFs) serve as a crucial signal to guide cell movement in processes such as wound healing, embryonic development, and cancer metastasis. However, the mechanism underlying cell electrotaxis remains poorly understood. A plausible hypothesis suggests that electrophoretic or electroosmotic forces may rearrange charged components of the cell membrane, including receptors for chemoattractants which induce asymmetric signaling and directional motility. This study aimed to explore the role of Transforming Growth Factor Beta (TGFβ) signaling in the electrotactic reaction of 3T3 fibroblasts. Our findings indicate that inhibiting canonical and several non-canonical signaling pathways originating from the activated TGF-β receptor does not hinder the directed migration of 3T3 cells to the cathode. Furthermore, suppression of TGF-β receptor expression does not eliminate the directional migration effect of 3T3 cells in the electric field. Additionally, there is no observed redistribution of the TGF-β receptor in the electric field. However, our studies affirm the significant involvement of Phosphoinositide 3-Kinase (PI3K) in electrotaxis, suggesting that in our model, its activation is likely associated with factors independent of TGFβ action.

## Introduction

The existence of endogenous electric fields in diverse anatomical locations within living organisms has been extensively documented across various research accounts [for a comprehensive overview, refer to the work of McCaig et al.( 2005)]. These electric fields emerge from polarized ion transport across epithelial tissues, resulting in the formation of a transepithelial potential. Following the disruption of the epithelial layer, such as by injury, this potential collapses at the center of the wound, while remaining stable in distal regions where ion transport is unaffected. This creates a voltage gradient, forming a direct current electric field (dcEF) with a vector parallel to the epithelial surface, and the wound center acting as the cathode (McCaig et al., 2009). Evidence increasingly highlights the pivotal role of electric signals in guiding cell migration during processes such as wound healing, embryonic development, and the metastasis of cancer (Djamgoz et al., 2001; McCaig et al., 2005; Zhao, 2009). This phenomenon is referred to as electrotaxis and is defined as an active directional movement toward the cathode or anode.

While electrotaxis is well-documented in various cell types, the molecular mechanisms employed by cells to sense electric fields remain largely elusive. It is hypothesized that electrophoretic or electroosmotic forces redistribute the charged components of the cell membrane, including receptors for chemoattractants. This redistribution could lead to a higher concentration of these receptors on the side of the cell facing the cathode (or anode), potentially driving asymmetric signaling that underlies directional movement (McCaig et al., 2005; Allen et al., 2013).

The role of chemoattractant receptor redistribution on the cell membrane during electrotaxis is critically important. In particular, the Epidermal Growth Factor (EGF) receptor has been identified as a key player in the electrotactic responses of various cell types, including corneal epithelial cells, keratinocytes, and breast cancer cells (Fang et al., 1999; Zhao et al., 2002; Pu et al., 2007). Small direct current electric fields not only upregulate the expression of EGF receptors but also induce their asymmetrical distribution within the cell membrane. Nevertheless, it has been proposed that the electrotaxis process depends on the redistribution of receptors specific to additional chemotactic factors such as Basic Fibroblast Growth Factor (bFGF), acetylcholine, Vascular Endothelial Growth Factor (VEGF), and Transforming Growth Factor Beta (TGF-β) (Orida and Poo, 1978; Zhao et al., 1996; Zhao et al., 1999; Zhao et al., 2012). However, the mechanism of receptor translocation does not sufficiently explain the nature of the first rapid reactions of cells to dcEF (Korohoda et al., 2000; Djamgoz et al., 2001; Zimolag et al., 2017). Another significant hypothesis posits that the mechanism underlying electrotaxis could stem from uneven activation of different ion channels within the cell membrane. Several ion channels have been implicated in the electrotactic responses of diverse cell types. These include calcium channels (Mycielska and Djamgoz, 2004), voltage-gated sodium channels (Djamgoz et al., 2001), voltage-gated potassium Kv1.2 channels (Zhang et al., 2016), inwardly rectifying potassium channels (Kir4.2) (Nakajima et al., 2015), and ion transporters such as Na, K-ATPase (NaKA) and Na+/H+ exchanger isoforms (NHE1 and 3) (Özkucur et al., 2011). Additionally, Liu et al. documented the activation of Na+ and K+ pumping modes of (Na,K)-ATPase by an oscillating electric field (Liu et al., 1990). Furthermore, large-scale screening methods identified several ion channel genes as potential key players in electrotaxis (Nakajima et al., 2015; Lasota et al., 2024).

In our previous investigation (Lasota et al., 2024), we demonstrated that in slowly migrating 3T3 cells, the response to a direct current electric field (dcEF) occurs quite swiftly, with initial signs becoming apparent after just 1 min of exposure. This strongly suggests that the primary mechanism governing the electrotactic response is linked to the activation of ion channels rather than the translocation of cell membrane receptors. Indeed, through comprehensive screening, we identified several ion channel genes as potentially involved in 3T3-cell electrotaxis. Notably, the electrotactic response of 3T3 cells was significantly reliant on inwardly rectifying potassium channels (Kir4.2). Since inhibiting the Kir4.2 channel only diminishes the directional movement of 3T3 cells for approximately 1–2 h, followed by the reappearance of electrotaxis, we proposed a biphasic mechanism for the electrotaxis of mouse 3T3 fibroblasts. According to this model, the activation of ion channels triggers the initial rapid cellular response to an electric field, while the redistribution of membrane receptors is responsible for the sustained directionality of cell movement over the long term. Our preliminary findings indicated that the prolonged response might be attributed to the relocation of the EGF receptor in the membrane of 3T3 cells. On the other hand, we have proven the involvement of TGFβ signaling in the electrotaxis of another cell type – human bronchial fibroblasts (Pavlenko et al., 2023). Building on these findings, the current study delves into the role of TGFβ signaling in the long-term reaction of 3T3 fibroblasts to an electric field.

## Materials and methods

### Cell culture

Mouse 3T3 fibroblasts (ATCC CRL-1658) were cultured in DMEM HG (Dulbecco’s Modified Eagle’s Medium High Glucose) medium (Sigma‒Aldrich, St. Louis, MO, United States), supplemented with 10% FBS (Fetal Bovine Serum) (Gibco, Waltham, MA, United States), penicillin (100 IU/mL), and streptomycin (100 μg/mL) (herein referred to as the ‘complete culture medium’) under standard conditions at 37°C and 5% CO_2_. Cells were passaged using 0.25% trypsin/EDTA (Gibco, Waltham, MA, United States) approximately every third day to maintain confluency below 80%.

### Cell seeding and application of electric field

To apply a direct current electric field (dcEF) to the cells, we used a custom electrotactic chamber based on a previously described protocol with minor modifications (Sroka et al., 2018). The scheme depicting the methodology of electrotaxis examination is presented in Supplementary Figure S1. We seeded 1×10^4^ cells onto a sterile 60 × 35 × 0.2 mm cover glass using 400 μL of complete culture medium, achieving a final cell density of approximately 2.5×10^3^ cells/cm^2^. Before the experiment, the second part of the observation chamber was assembled using another cover glass and two additional 60 × 10 × 0.2 mm glass pieces, connected with double-sided adhesive tape (tesa SE, Hamburg, Germany). This assembly, once completed, was filled with fresh complete culture medium or serum-free medium and inserted into an external electrotactic chamber made of PVC. Ag|AgCl electrodes (each 6 cm^2^) immersed in PBS were connected to a power supply. These electrodes were linked to the observation chamber using salt bridges made of glass pipes filled with agar (2% in 0.5 M KCl). Applying a dcEF with an intensity of 3 V/cm completed the setup. In the case of experiments without a dcEF, cells were seeded onto 24-well plates with glass bottoms (Eppendorf, Hamburg, Germany) in the quantity of 5 × 10^3^ per well.

### Registration and analysis of cell migration

Time-lapse imaging was conducted using integrated modulation contrast (IMC). For electrotaxis studies, a Leica DM IL LED microscope (Leica, Wetzlar, Germany) equipped with a Moticam 3.0 camera controlled by Motic Images Plus 3.0 software (both Motic, Xiamen, China) was used. For the registration in isotropic conditions (w/o dcEF), we employed a Leica DMI6000B motorized microscope with a Leica DFC360FX camera operated via LAS X 3.4 software (all Leica, Wetzlar, Germany). A temperature of 37°C was maintained inside the observation chamber using an environmental chamber and a heating unit. To offset the lack of CO_2_ control, HEPES buffer (15 mM) was added to the complete culture medium before each recording session. The images were captured every 5 min for 4 h (with the cathode on the right-hand side).

Analysis of single-cell migration was performed using Hiro 1.0.0.4 software (Krecioch et al., 2015; Sroka et al., 2016; Sroka et al., 2018), where cell migration trajectories were constructed from manually identified cell centroids. To obtain circular diagrams, the initial point of each trajectory was placed in the origin of the coordinate system. The following quantitative parameters were calculated: (a) the speed of cell migration (µm/min)—the total length of the cell trajectory divided by the time of recording; (b) cell displacement (µm)—the length of the line segment from the first to the last position of a cell; (c) average directional cosine γ—γ is the angle formed by the line that connected the initial point of trajectory to subsequent cell positions and the *X*-axis (parallel to the vector of EF); (d) CME (Coefficient of Movement Efficiency)—representing the ratio of displacement to the total length of the cell trajectory. It approaches 1 when cell migration is persistent and decreases toward 0 when the direction of movement changes frequently.

### Pharmacological modification of signaling pathways

To investigate the role of TGFβ signaling in the electrotaxis of 3T3 fibroblasts, we utilized inhibitors targeting both the canonical and noncanonical TGF-β pathways. Specifically, to block the canonical Smad-dependent signaling pathway, SB431542 (10 μM) was introduced into the complete culture medium during the preparation of the chamber. This inhibitor was applied 30 min prior to recording cell migration and was present throughout the duration of the experiment. To inhibit the noncanonical signaling pathways, the following inhibitors were added: U0126 (10 μM) for MEK1/2, SB203580 (10 μM) for p38, Y-27632 (10 μM) for ROCK, and LY-294002 (20 μM) for PI3K, following the protocol for the SB431542 inhibitor (all inhibitors from Sigma-Aldrich, St. Louis, MO, United States). In experiments without the presence of serum, the serum-free medium was additionally supplemented with TGF-β1 at a concentration of 5 ng/mL.

### Knockdown of the TGF-β receptor gene

To verify the involvement of Tgfbr2 in the electrotaxis of 3T3 fibroblasts, the receptor was knocked down with a specific siRNA. Briefly, 5 × 10^4^ cells/well were seeded into a 12-well plate the day before. Cells were transfected with 25 nM siRNA targeting mouse Tgfbr2 (#AM16708, siRNA ID: 64894, Ambion, Austin, TX, United States) or non-targeting control (#AM4611, Ambion) using DharmaFECT 1 (Dharmacon, Lafayette, CO, United States) as the transfection reagent. The procedure was performed according to the manufacturer’s instructions. Approximately 24 h after transfection, cells were harvested and seeded into an electrotactic chamber to allow for adherence. Electrotaxis was analyzed 24 h later as described above.

### Western Blot analysis

Cells transfected with siRNA were harvested 48 h after transfection and lysed with RIPA lysis buffer (Sigma-Aldrich) supplemented with protease and phosphatase inhibitors (Thermo Fisher Scientific, Waltham, MA, United States). The lysates were then sonicated (3 × 10 s) with intermediate incubations on ice and centrifuged at 16.000 *g* for 10 min at 4°C to isolate the protein fraction. Protein concentration was measured using a BCA assay (Thermo Fisher Scientific) according to the manufacturer’s instructions. Samples were then mixed with 4x Loading Protein Buffer Plus (Eurx, Gdańsk, Poland) and boiled at 95°C for 5 min 10 μg protein samples were separated by SDS-PAGE using Miniprotean TGX Gel, 4%–15% (Bio-Rad, Hercules, CA, United States) and transferred onto a polyvinylidene fluoride (PVDF) membrane using a Trans-Blot Turbo Transfer Pack Midi Format 0. 2 μM PVDF (Bio-Rad) in a semi-dry transfer at 25 V, 1.3 A for 7 min in the Trans-Blot Turbo Transfer System (Bio-Rad). Membranes were incubated in 3% BSA in TBST (0.05% (v/v) Tween 20 in Tris-buffered saline) for 90 min to block nonspecific binding sites. The membranes were then incubated with primary antibodies against TGFBR2 (#GTX55814, GeneTex, Irvine, CA, United States) or GAPHD (#MA5-15738, Invitrogen/Thermo Fisher Scientific) as a loading control overnight at 4°C with gentle shaking. The next day, the membranes were washed three times with TBST and incubated with horseradish peroxidase (HRP)-conjugated secondary antibodies: goat anti-rabbit IgG, horse anti-mouse IgG (#7074 and #7076, respectively, both from Cell Signaling Technology, Danvers, MA, United States) in 3% BSA in TBST for 50 min at room temperature. After washing the membranes 3 times with TBST, the signal was detected with the chemiluminescent HRP substrate (Merck, Rahway, NJ, United States) in a ChemiDoc XRS + imager (BioRad). Densitometric analysis was performed using Quantity One software (Bio-Rad).

### TGF-β receptor distribution

3T3 fibroblasts were seeded into a 24-well plate at a density of 2.5×10^4^ cells per well and incubated overnight under standard conditions. The next day, cells were transfected in accordance with the manufacturer’s protocol (Thermo Fisher Scientific, Waltham, MA, United States) using 0.75 µL of Lipofectamine 3000 reagent and 0.5 µg of a plasmid vector encoding the TGFBR1 (ALK5) receptor fused to an mEmerald Green fluorescent protein (Addgene, Watertown, MA, United States, #62751) for each well. Twenty-four hours post-transfection, cells were reseeded onto 60 × 35 × 0.2 mm glass slides for the electrotaxis chamber as outlined in the preceding section. Given the rapid reduction in the expression of the protein, transient transfection was scheduled 48 h before the planned imaging of the receptor redistribution. Imaging was done utilizing a Leica DMI8 inverted fluorescence microscope equipped with a DFC7000 GT monochromatic CCD camera, dry HCX APO U-V-I 40x/0.75 DRY UV objective, a GFP-optimized filter set (all Leica, Wetzlar, Germany), and a CoolLED pE-4000 LED illuminator (CoolLED Ltd., Andover, Great Britain). Following the application of the electric field, as previously described, images of fluorescent cells across various fields of view were recorded. These images were then processed with ImageJ Fiji software (National Institute of Health, Bethesda, MD, United States) (Schindelin et al., 2012), i.e., the images were shading corrected and used for designation of plot profiles along lines with a thickness of 5 pixels, with the intention of reducing noise.

### Statistical analysis

The statistical significance of variances in cell migration speed, cell displacement, and CME was evaluated using the Student’s t-test. In contrast, for directionality, these differences were examined using a non-parametric U–Mann–Whitney test due to the nature of this parameter and its inherent lack of a normal distribution (confirmed by the Shapiro-Wilk test). All statistical analyses were conducted using GraphPad Prism version 8.0.1 (GraphPad Software, Boston, MA, United States), with a threshold for statistical significance set at *p* < 0.05.

## Results

### The impact of inhibiting canonical and non-canonical TGF-β signaling pathways on 3T3 cell electrotaxis in the presence of a serum

**FIGURE 1:**
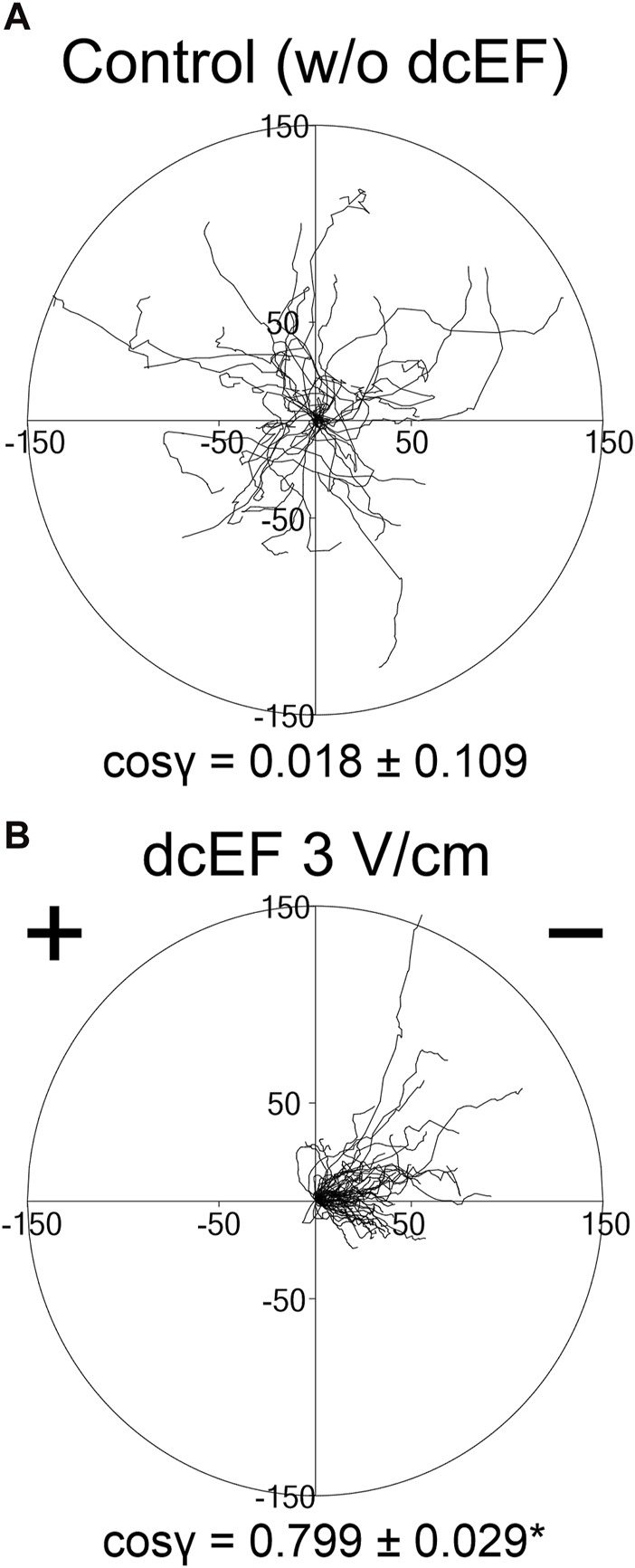
Movement of 3T3 fibroblasts in an isotropic environment and under the influence of a direct-current electric field (dcEF) of physiological strength. Circular diagrams depict the combined trajectories of individual cell migration **(A)** in the absence of dcEF and **(B)** with a dcEF of 3 V/cm. The starting point for each trajectory (formed by tracking 48 consecutive positions of the cell centroid, recorded every 5 min) was repositioned to the origin of the coordinate system. The dcEF cathode, when applicable, is situated to the right in the illustration. Scale is provided in micrometers (μm). The directional cos γ values below (referring to the directionality of the cell migration), are provided as the mean (for the cell population; *n* = 30 and 60, respectively) ± the standard error of the mean (SEM). *Statistically significant difference between compared conditions (dcEF 3 V/cm vs. w/o dcEF) (*p* < 0.05).

As previously communicated, 3T3 fibroblasts in a medium with 10% FBS exhibit robust electrotactic activity (Lasota et al., 2024). Indeed, as shown in Figure 1A, in the absence of an electric field, the 3T3 cells displayed random movements (cos γ = 0.018 ± 0.109). However, following the application of an electric field (3 V/cm), the cell migration became distinctly directional, with cells moving toward the cathode (cos γ = 0.799 ± 0.029) (Figure 1B).

**FIGURE 2:**
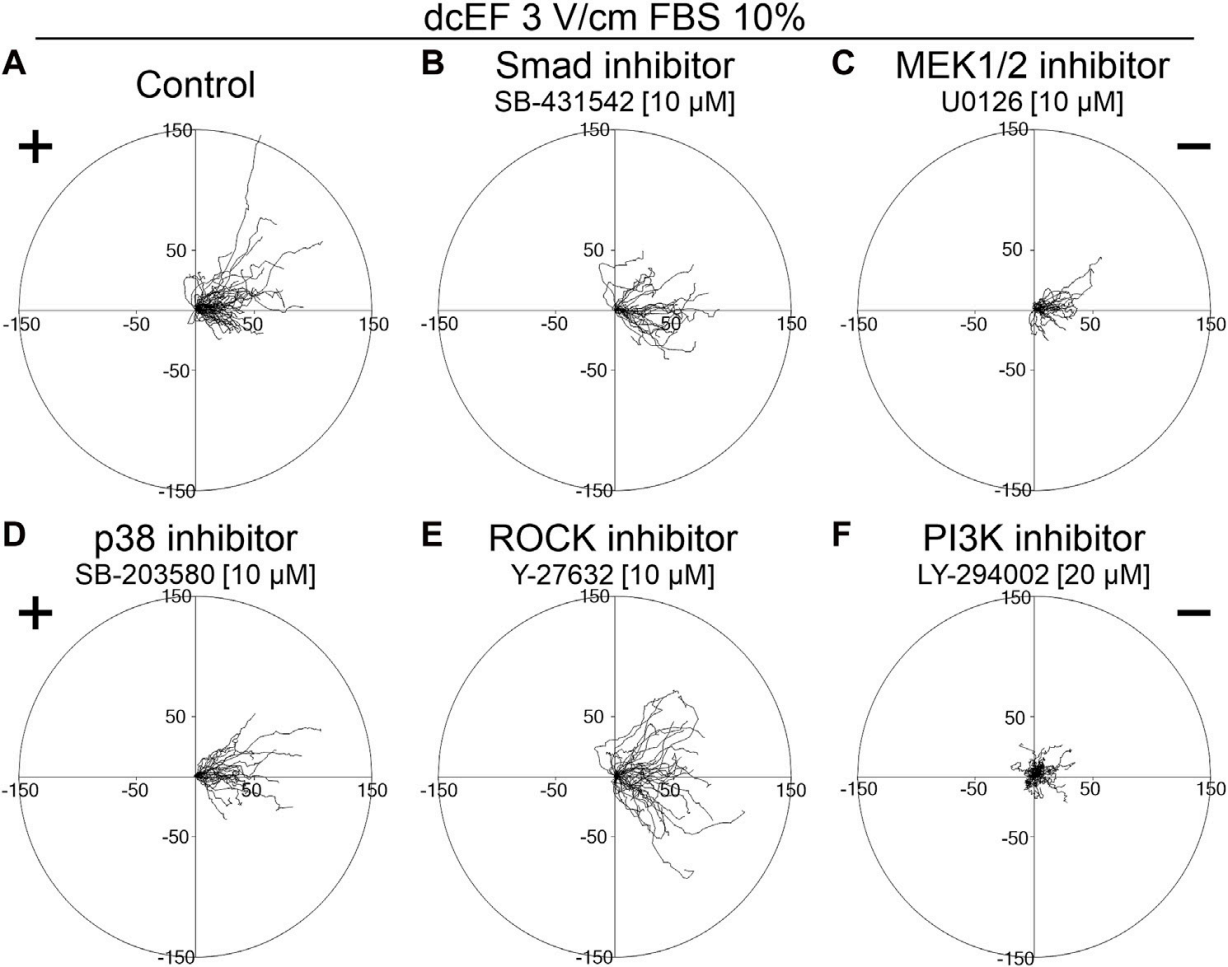
The role of TGF-β signaling in the electrotaxis of 3T3 fibroblasts examined in the presence of a serum. Circular diagrams illustrating composite cell trajectories under various conditions: **(A)** control conditions (dcEF 3 V/cm, FBS 10%), and in the presence of **(B)** Smad inhibitor - SB-431542 (10 μM), **(C)** MEK1/2 inhibitor - U0126 (10 μM), **(D)** p38 inhibitor - SB-203580 (10 μM), **(E)** ROCK inhibitor - Y-27632 (10 μM), and **(F)** PI3K inhibitor - LY-294002 (20 μM). The methodology for constructing and displaying these diagrams follows that of the previous figure (refer to Figure 1).

**FIGURE 3:**
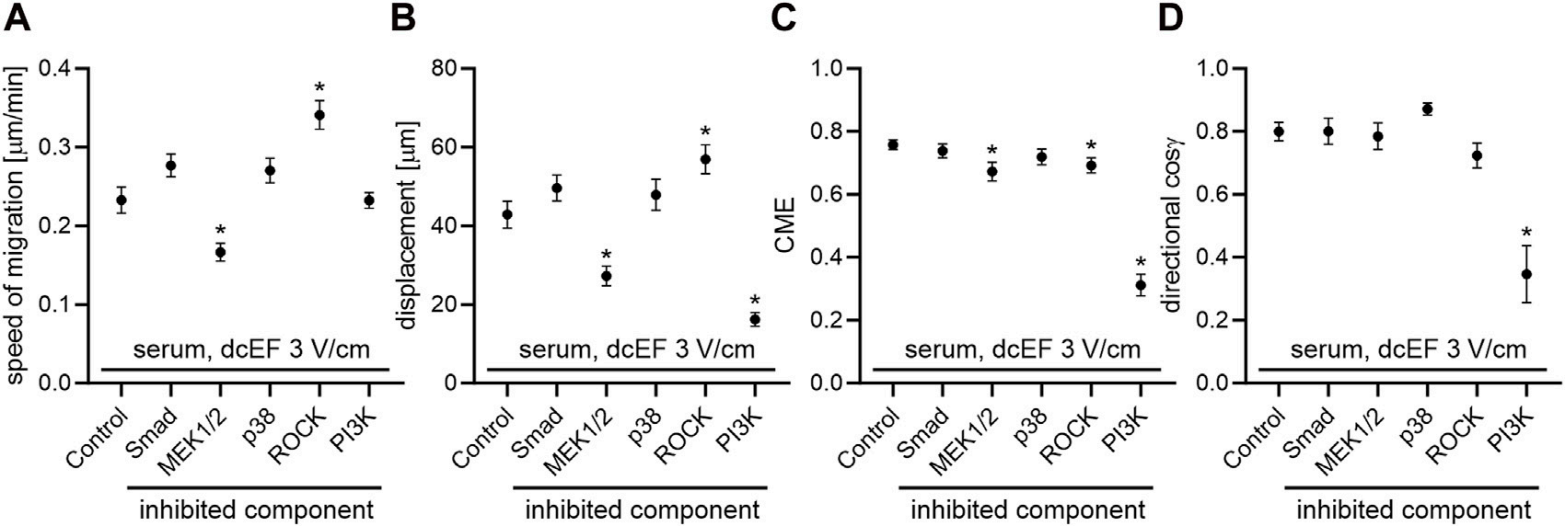
The impact of TGF-β signaling inhibition on the electrotaxis of 3T3 fibroblasts in the presence of a serum – quantitative analysis. **(A)** Speed of cell migration, **(B)** displacement, **(C)** coefficient of movement efficiency (CME), and **(D)** directionality of cell migration (presented as the average directional cosine γ), presented as the mean (for the cell population) ± standard error of the mean (SEM). The number of cells (*n*) is detailed in Table 1. *Statistically significant differences compared to the control (dcEF 3 V/cm, 10% FBS) (*p* < 0.05).

To determine the role of TGFβ in directing the migration of 3T3 cells in an electric field, we assessed the effects of inhibiting both canonical and non-canonical signaling pathways activated by TGFβ, using specific pharmacological inhibitors at concentrations validated in our previous studies (Paw et al., 2021; Pavlenko et al., 2023). As presented in Figure 2 and Figure 3D, inhibition of the canonical Smad-dependent pathway with SB431542 did not alter the electrotaxis of 3T3 cells. Similarly, inhibitors of noncanonical TGFβ signaling pathways, specifically MEK1/2 (U0126), p38 (SB203580), and ROCK (Y-27632), did not affect the directional migration of 3T3 cells in the electric field. However, the PI3K signaling pathway stood out as its inhibition with LY-294002 significantly reduced the directional cosine from 0.799 ± 0.029 under the control conditions to 0.347 ± 0.092 with the inhibitor present (Figure 3D; Table 1), pointing to a critical role of PI3K in migration directionality. Notably, while U0126 (MEK1/2 inhibitor) and Y-27632 (ROCK inhibitor) significantly influenced the speed of migration and cell displacement, suggesting the importance of these signaling pathways for motile activity, they did not affect the directionality of cell movement (Figure 3; Table 1). A similar effect of these inhibitors on the migration of 3T3 cells was found under control conditions without the application of an electric field (Supplementary Figure S2).

**TABLE 1:** The impact of TGF-β signaling inhibition on the electrotaxis of 3T3 fibroblasts in the presence of a serum.

	Serum, dcEF 3 V/cm					
	Control (serum, dcEF 3 V/cm) (n = 60)	Smad inh.- SB-431542 (10 μM) (n = 30)	MEK 1/2 inh.- U0126 (10 μM) (n = 30)	p38 inh.- SB-203580 (10 μM) (n = 30)	ROCK inh.- Y-27632 (10 μM) (n = 40)	PI3K inh.- LY-294002 (20 μM) (n = 30)
Speed of migration [μm/min]	0.233 ± 0.017	0.277 ± 0.015	0.166 ± 0.011	0.271 ± 0.016	0.341 ± 0.018^a^	0.233 ± 0.010
Displacement [μm]	42.879 ± 3.391	49.657 ± 3.333	27.285 ± 2.493^a^	47.910 ± 4.025	56.967 ± 3.693^a^	16.227 ± 1.754^a^
CME	0.758 ± 0.015	0.739 ± 0.022	0.673 ± 0.030^a^	0.720 ± 0.025	0.692 ± 0.024^a^	0.312 ± 0.035^a^
Cosine γ	0.799 ± 0.029	0.800 ± 0.042	0.785 ± 0.043	0.871 ± 0.020	0.724 ± 0.040	0.347 ± 0.092^a^

^a^ Statistically significant differences relative to control (serum, direct current electric field 3 V/cm) (*p* < 0.05).

The presented results underscore that among the TGFβ-activated signaling pathways we explored, only the PI3K-dependent pathway is crucial for electrotaxis. Nonetheless, given that this pathway can also be activated by additional factors within the FBS (e.g., EGF), we conducted additional experiments to investigate the impact of the tested inhibitors on the electrotaxis of 3T3 cells in a serum-free medium, supplemented with exogenously added TGFβ.

### The impact of inhibiting canonical and non-canonical TGF-β signaling pathways on 3T3 cell electrotaxis in a serum-free media

**FIGURE 4:**
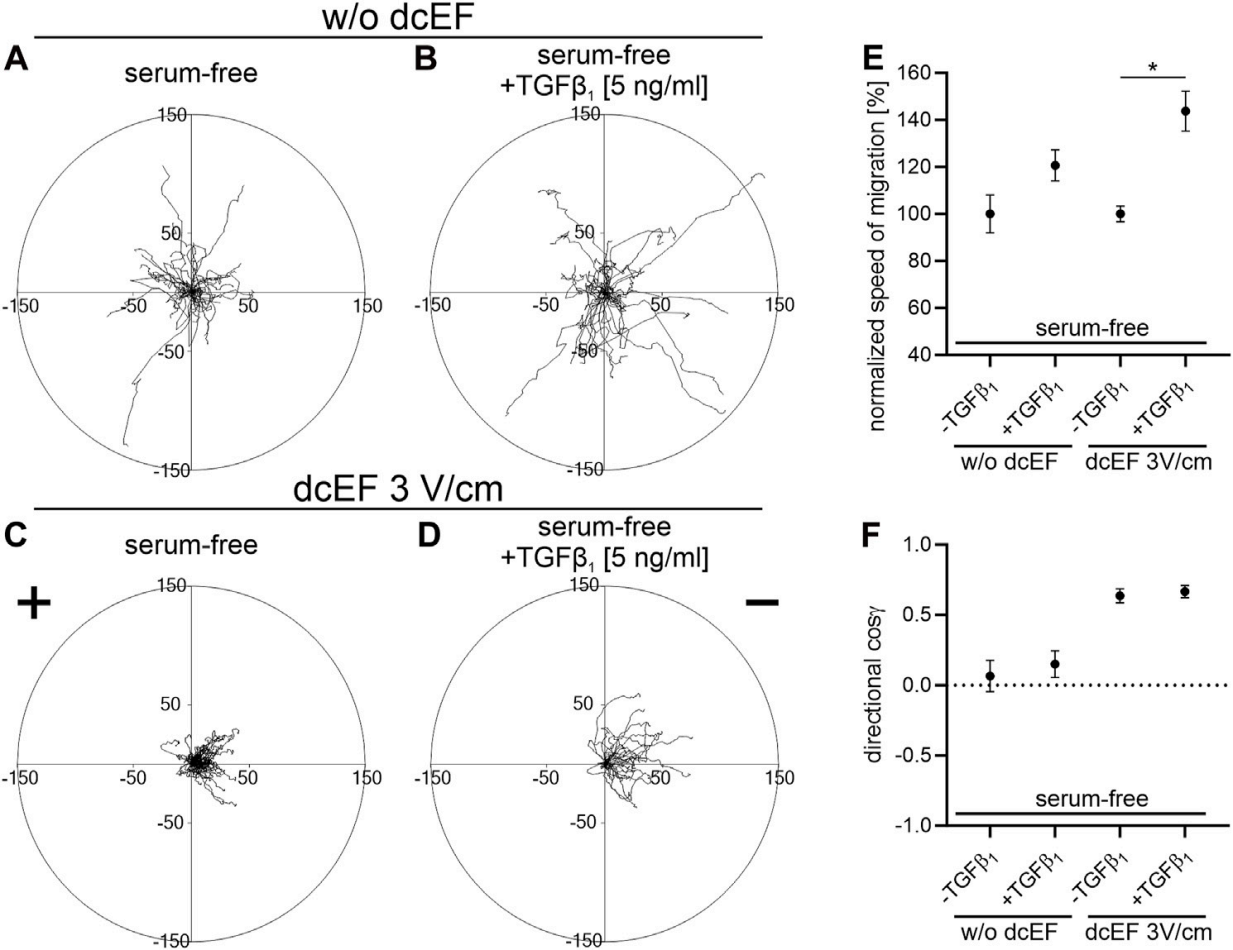
The impact of TGF-β1 on the electrotaxis of 3T3 fibroblasts in serum-free media. **(A–D)** Circular diagrams illustrate the combined trajectories of individual cell migrations **(A,B)** without dcEF and **(C,D)** in the presence of a dcEF of 3 V/cm. All experiments were performed in serum-free conditions, with the culture media in **(B,D)** further enriched with TGF-β1 (5 ng/mL). The approach for chart creation and presentation is consistent with that described previously (see Figure 1). **(E,F)** Graphs showing quantitative parameters summarizing the effect of TGF- β1 (5 ng/mL) addition to the culture medium. **(E)** Speed of cell migration normalized to corresponding control (-TGFβ_1_) and **(F)** directionality of cell migration (shown as the average directional cosine γ), presented as the mean (for the cell population) ± the standard error of the mean (SEM). The number of cells (n) is 30, 30, 60, and 30, respectively. *Statistically significant differences compared to the corresponding control (serum-free, w/o TGF-β1) (*p* < 0.05).

In serum-free conditions, 3T3 cells demonstrated a distinct electrotactic response, migrating toward the cathode in an electric field of 3V/cm (Figures 4A,C). The directional cosine in these conditions was only marginally lower than in the presence of 10% FBS (0.636 ± 0.050 and 0.799 ± 0.029, respectively). However, both the speed of cell migration and their final displacement were reduced in the absence of serum compared to conditions with 10% serum (0.169 ± 0.006* μm/min vs. 0.233 ± 0.017 μm/min and 20.098 ± 1.364* μm vs. 42.879 ± 3.391 μm, respectively). Notably, adding exogenous TGFβ (5 ng/mL) did not alter the directionality of cell movement (Figure 4F), yet significantly enhanced their migration speed and final displacement (Figure 4E; Table 2).

**TABLE 2:** The impact of TGF-β signaling inhibition on the electrotaxis of 3T3 fibroblasts in the serum-free medium containing TGF-β_1_.

	Serum-free + TGFβ_1_ (5 ng/mL), dcEF 3 V/cm				
	Control (serum-free + TGFβ1 5 ng/mL, dcEF 3 V/cm) (n = 30)	Smad inhibitor - SB-431542 (10 μM) (n = 30)	p38 inhibitor - SB-203580 (10 μM) (n = 40)	ROCK inhibitor - Y-27632 (10 μM) (n = 35)	PI3K inhibitor- LY-294002 (20 μM) (n = 30)
Speed of migration [μm/min]	0.243 ± 0.015	0.233 ± 0.014	0.251 ± 0.014	0.190 ± 0.012^a^	0.191 ± 0.013^a^
Displacement [μm]	35.990 ± 3.265	41.918 ± 3.457	33.711 ± 3.912	27.825 ± 2.853	19.756 ± 3.029^a^
CME	0.608 ± 0.030	0.736 ± 0.029^a^	0.518 ± 0.039	0.586 ± 0.035	0.396 ± 0.044^a^
Cosine γ	0.666 ± 0.045	0.767 ± 0.035	0.517 ± 0.072	0.539 ± 0.073	0.506 ± 0.081

^a^ Statistically significant differences relative to the control (serum-free + Transforming Growth Factor Beta 1, direct current electric field 3 V/cm) (*p* < 0.05).

**FIGURE 5:**
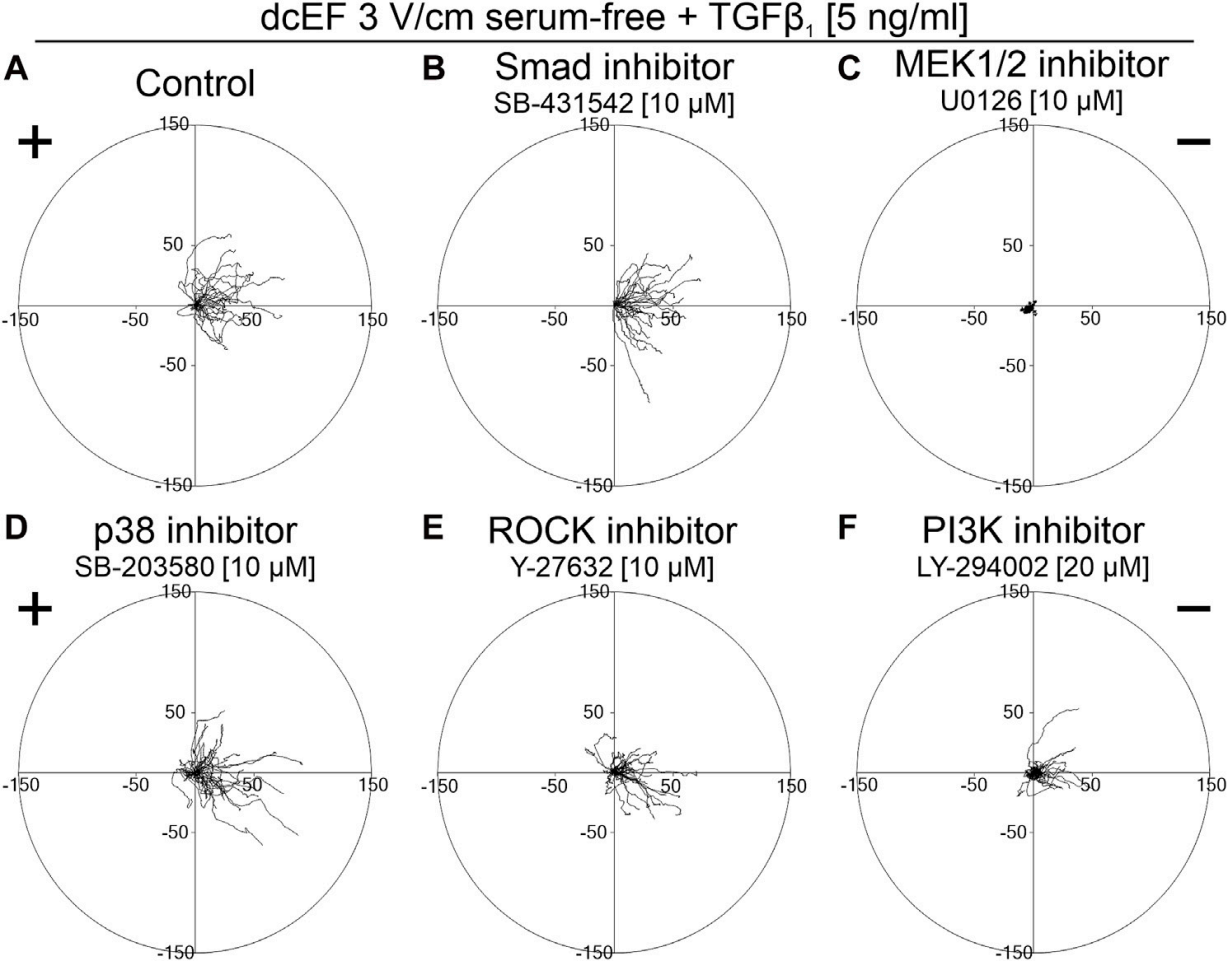
The role of TGF-β signaling in the electrotaxis of 3T3 fibroblasts examined in the serum-free medium containing TGF-β_1_. Circular diagrams illustrating composite cell trajectories under various conditions: **(A)** control conditions (dcEF 3 V/cm, serum-free, TGF-β_1_ (5 ng/mL)), and in the presence of **(B)** Smad inhibitor - SB-431542 (10 μM), **(C)** MEK1/2 inhibitor - U0126 (10 μM), **(D)** p38 inhibitor - SB-203580 (10 μM), **(E)** ROCK inhibitor - Y-27632 (10 μM), and **(F)** PI3K inhibitor - LY-294002 (20 μM). The methodology for constructing and displaying these diagrams follows that of Figure 1.

**FIGURE 6:**
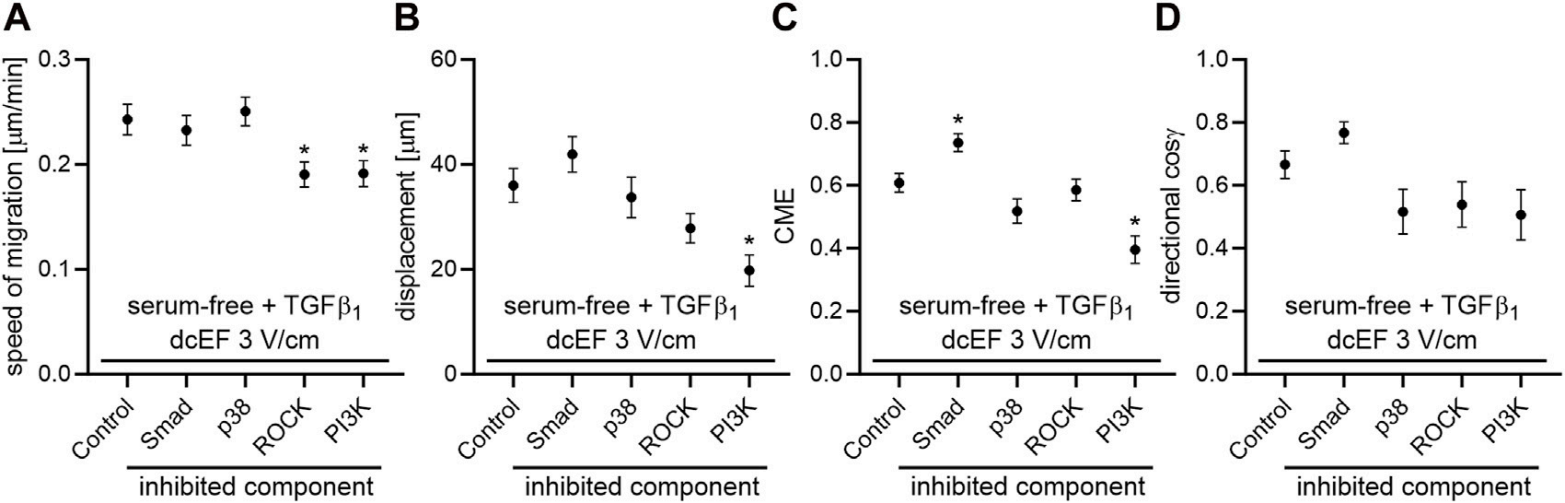
The impact of TGF-β signaling inhibition on the electrotaxis of 3T3 fibroblasts in the serum-free medium containing TGF-β_1_ – quantitative analysis. **(A)** Speed of cell migration, **(B)** displacement, **(C)** coefficient of movement efficiency (CME) and **(D)** directionality of cell migration (presented as the average directional cosine γ), presented as the mean (for the cell population) ± the standard error of the mean (SEM). The number of cells (*n*) is detailed in Table 2. *Statistically significant differences compared to the control (dcEF 3 V/cm, serum-free, TGF-β_1_ (5 ng/mL)) (*p* < 0.05).

As depicted in Figure 5 and Figure 6, in serum-free media at the presence of TGFβ (5 ng/mL), SB431542, an inhibitor targeting the canonical Smad-dependent signaling pathway, did not impact the electrotaxis of 3T3 cells. Similarly, inhibitors of noncanonical TGFβ signaling pathways such as p38 (SB203580) and ROCK (Y-27632) exhibited no discernible effect on the directional migration of 3T3 cells in the electric field. Intriguingly, even the inhibition of the PI3K signaling pathway did not significantly diminish electrotaxis under these experimental conditions. It is worth emphasizing that the inhibitor of MEK1/2 (U0126) completely halted the migration of 3T3 cells (Figure 5C). It is important to note that data from non-migrating cells were excluded from further analysis, as movement parameters could not be determined. The effects of all these inhibitors on the migration of 3T3 cells in the absence of an electric field are detailed in Supplementary Figure S3.

The obtained results suggest that although the PI3K pathway emerges as a crucial component in the response of 3T3 cells to an electric field, its activation in this context does not appear to be directly related to TGFβ. Instead, it is likely associated with other factors present in the serum that stimulate this pathway.

Given that our findings do not affirm a substantial role of TGFβ in driving electrotaxis, we endeavoured in subsequent experiments to validate the hypothesis concerning the translocation of the TGFβ receptor within the cell membrane under the influence of an applied electric field.

### TGFβ receptor redistribution in an electric field and its implication in 3T3 fibroblast electrotaxis

**FIGURE 7:**
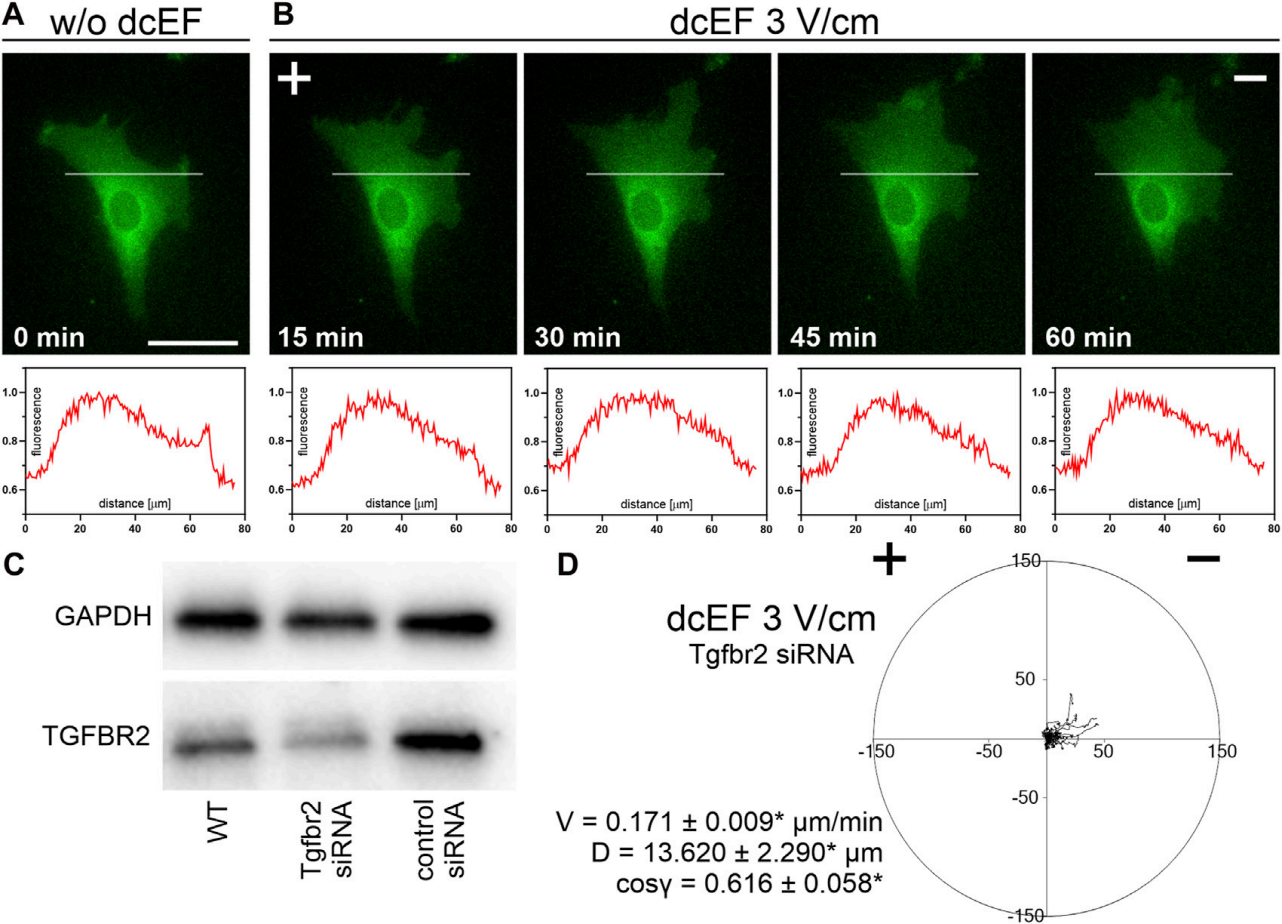
The distribution and role of TGF receptors during the electrotaxis of 3T3 fibroblasts. **(A,B)** Fluorescence images of cells expressing TGFBR1 (ALK5) fused to a fluorescent protein (green) both in the absence of an electric field **(A)** and following a defined period of stimulation by a dcEF of 3 V/cm **(B)**. The scale bar in **(A)**, representing 50 μm, applies to all images. When applicable, the dcEF’s cathode is positioned on the right side of the field of view. Each image is accompanied by a corresponding plot profile that illustrates the normalized intensity of green fluorescence along the indicated white line. **(C)** Western blot analysis demonstrates reduced levels of TGFBR2 protein in cells transfected with specific siRNA compared to wild-type cells and those transfected with non-targeting siRNA. **(D)** A circular diagram shows the combined migration trajectories of individual cells (with Tgfrb2 silenced) in a dcEF of 3 V/cm. The approach for diagram creation and presentation is consistent with that described in a prior figure (see Figure 1). Quantitative parameters: V - speed of cell migration, D – displacement and directional cos γ (referring to the directionality of cell migration), are provided as the mean (for the cell population *n* = 30) ± the standard error of the mean (SEM). *Statistically significant differences compared to the control (dcEF 3 V/cm, 10% FBS) (*p* < 0.05).

To test this hypothesis, we investigated the kinetics of TGFβR redistribution within the cell membrane of the 3T3 cells exposed to a direct current electric field (dcEF) of 3 V/cm, focusing on potential receptor displacement within a specified time frame. The results, illustrated in Figures 7A,B, reveal that after 15–60 min of exposure to a 3 V/cm dcEF, there was no detectable accumulation of TGFβ receptors on the cathode- or anode-facing side of the cell.

As this result aligned with our earlier observations, we proceeded to investigate the impact of TGFβ receptor silencing (Figure 7C) on the electrotaxis of 3T3 fibroblasts in a medium containing 10% FBS. As depicted in Figure 7D, the silencing of the receptor led to only a minor reduction in the average directional cosine, with values slightly lower than the control (cos γ = 0.616 ± 0.058* compared to 0.799 ± 0.029).

## Discussion

The understanding of the intricacies of electrotaxis remains incomplete, with two main hypotheses competing to explain this phenomenon. The first hypothesis predominantly concentrates on the asymmetrical activation of ion channels within the cell membrane, triggered by hyperpolarization of the cell membrane at the anodal side and depolarization at the cathodal side of the cell in response to an external electric field, or induced by mechanical effects from dcEF (Mycielska and Djamgoz, 2004; Gao et al., 2011; Allen et al., 2013). Although effective in elucidating the initial rapid responses of cells to dcEF (Korohoda et al., 2000; Djamgoz et al., 2001), this hypothesis does not effectively explain the unclear mechanism responsible for the asymmetrical activation of these ion channels by the electric field (Mycielska and Djamgoz, 2004).

On the other hand, the second plausible hypothesis posits that electrophoretic or electroosmotic forces alter the distribution of charged components on the cell membrane, including receptors for chemoattractants. It is proposed that an increased concentration of these receptors on the side of the cell facing the cathode (or anode) triggers asymmetric signaling and directional motility (McCaig et al., 2005; Allen et al., 2013). However, this hypothesis does not fully explain the swift initial reactions of cells to dcEF (Korohoda et al., 2000; Djamgoz et al., 2001). Nevertheless, it should be acknowledged that both mechanisms, whether activation of the ion channel or receptor redistribution, are not mutually exclusive and may synergistically contribute to the process of electrotactic migration.

In our previous study (Lasota et al., 2024), we demonstrated the rapid response of 3T3 cells to a direct current electric field (dcEF), manifesting within the first minute of exposure. This response was notably reliant on inwardly rectifying potassium channels, particularly Kir4.2. Inhibiting these channels led to a transient reduction in the directional movement of 3T3 cells lasting approximately 1–2 h, followed by a reappearance of electrotaxis. We proposed a biphasic mechanism for the electrotaxis of mouse 3T3 fibroblasts, suggesting that ion channel activation underlies the initial rapid cellular response to an electric field, while the long-term maintenance of directional movement is facilitated by the redistribution of membrane receptors. Notably, we observed a distinct accumulation of EGF receptors on the side of the cell facing the cathode after 30–60 min in a dcEF (3 V/cm). Moreover, inhibition of the EGF receptor signaling pathway partially impeded the electrotaxis of 3T3 fibroblasts. Given that the relocation of EGF receptors has been established as a crucial factor in electrotaxis for various cell types (Fang et al., 1999; Zhao et al., 1999; Zhao et al., 2002), we postulated that the EGF pathway may underlie the sustained electrotactic response in 3T3 cells.

However, beyond the EGF receptor, the importance of receptor redistribution in the electrotaxis process has been suggested for additional chemotactic factors (Orida and Poo, 1978; Zhao et al., 1996; Zhao et al., 1999; Zhao et al., 2012), including Transforming Growth Factor β (TGFβ). Zhao et al. (1996), Zhao et al. (1999) reported that TGFβ restored cathodal-directed migration of corneal epithelial cells (CECs) under serum-free conditions. However, these studies did not examine how the inhibition of TGFβ-activated signaling pathways might affect CEC electrotaxis.

Nevertheless, our research (Pavlenko et al., 2023) has shown that adult human bronchial fibroblasts respond to external electric fields by orienting themselves perpendicular to the field lines and exhibit efficient electrotaxis toward the anode. These processes depended on both canonical and non-canonical TGF-β pathways. This observation suggested the possibility that the electrotaxis of 3T3 fibroblasts might also be dependent on TGFβ.

Transforming growth factor beta (TGF-β) is a cytokine known to activate a variety of signaling pathways, leading to diverse biological effects including the stimulation of cell migration (Hao et al., 2019; Tie et al., 2022; Massagué and Sheppard, 2023). Within TGF-β-induced migratory signaling, two main types of pathways are recognized: canonical and noncanonical. The canonical pathways are Smad-dependent, where TGF-β receptor activation leads to phosphorylation and activation of Smad2 and Smad3 proteins. These phosphorylated Smad proteins then form a complex with Smad4 to move into the cell nucleus, regulating the expression of genes associated with cell migration (Costanza et al., 2017; Tie et al., 2022). On the other hand, the noncanonical MAPK pathway, comprising ERK, JNK, and p38 MAPK, can be activated by TGF-β, affecting cytoskeletal dynamics, cell adhesion, and migration. Additionally, noncanonical pathways include the Rho GTPase pathway (involving RhoA, Rac1, and Cdc42) and the PI3K/Akt pathway, each playing a role in cell migration regulation (Ridley et al., 2003; Costanza et al., 2017; Tie et al., 2022). Nevertheless, there is a lack of genetic evidence and a structural foundation supporting the notion that MAPKs and PI3K directly act as mediators in TGF-β receptor signaling. Moreover, these pathways already have established agonists which are commonly present in the microenvironment of TGF-β target cells *in vivo*, prompting inquiries into the significance of TGF-β as an activator of these pathways (Tie et al., 2022).

Unexpectedly, in our hands, inhibiting both the canonical and several non-canonical signaling pathways from the activated TGF-β receptor did not hinder the directional migration of 3T3 cells toward the cathode. Notably, the only statistically significant reduction in electrotaxis occurred with the inhibition of PI3K kinase (Figure 2; Figure 3).

This observation aligns with previous reports highlighting the essential role of PI3K activity in electrotaxis. For instance, Zhao et al. (2006) found that the genetic disruption of phosphatidylinositol-3-OH kinase-γ (PI(3)K γ) diminished electric-field-induced signaling and abolished electrotaxis of healing epithelium in response to electric signals. Similarly, Meng et al. (2011) demonstrated the significance of the PI3K pathway in the electrotaxis of neural progenitor cells (NPCs) driven by electric fields in the presence of growth factors.

However, given that PI3K activity can also be induced by additional factors in FBS (such as EGF) (Zhao et al., 2006; Meng et al., 2011), subsequent experiments were conducted to examine the influence of the tested inhibitors on the electrotaxis of 3T3 cells in a serum-free medium, supplemented with exogenously added TGFβ. This approach aimed to minimize the influence of PI3K-activating chemoattractants other than TGFβ. In this simplified model, none of the inhibitors used, including LY-294002 (PI3K inhibitor), affected electrotaxis. This suggests that while PI3K plays a crucial role in the induction of directional migration of 3T3 cells in an electric field (as evidenced by the inhibition of electrotaxis in the presence of FBS after the addition of LY-294002), its activation in our experiments was probably related to the action of factors other than TGFβ present in the serum. Literature reports, including our own (Zhao et al., 1996; Zhao et al., 1999; Meng et al., 2011; Lasota et al., 2024), suggest EGF as a possible activator.

The negligible effect of TGF signaling on the electrotaxis of 3T3 fibroblasts is further confirmed by the observation that the addition of TGFβ in serum-free conditions does not substantially influence the directionality of cell movement (Figure 4). However, it is important to note that the presence of TGFβ does induce a significant increase in the velocity of 3T3 cell movement (Figure 4E).

Our findings of TGFβ′s limited role in the regulation of electrotaxis in 3T3 cells were further supported by experiments investigating the redistribution of TGFβ receptors in an electric field. After 60 min in a dcEF of 3 V/cm, we did not observe any discernible accumulation of TGFβ receptors on the cathode-facing side of the cells (Figure 7B). This is in line with Zhao et al. (1999), who also reported no clear asymmetry in TGFβ receptor distribution under a 1.5 V/cm electric field, even after 3 h. Substantial accumulation was only noticeable after 12–16 h in the electric field. Although TGFβ partially restored cathodal-directed migration of corneal epithelial cells (CECs) in a serum-free medium over a 5-h period, the mechanism responsible for the observed electrotaxis remained unclear (Zhao et al., 1999). Interestingly, the relatively low electroosmotic mobilities of the TGFβ receptor also align with the theoretical model proposed by Sarkar et al. (2019). The non-involvement of TGFβ in the regulation of electrotaxis in 3T3 fibroblasts was further validated through experiments where the silencing of TGFβ receptors in 3T3 cells led to only a marginal reduction in the average directional cosine (Figure 7D).

By systematically inhibiting both canonical and non-canonical TGF-β signaling pathways, we demonstrated that TGF-β signaling does not play a major role in the directional migration of 3T3 fibroblasts in an electric field. The use of both serum-free and FBS-supplemented conditions further strengthens the reliability of our findings. Simultaneously, our study highlights significant insights into the role of PI3K kinase in the electrotaxis of 3T3 fibroblasts. However, our *in vitro* model has limitations, as it may not fully replicate the complex *in vivo* environment. Moreover, the findings, based on the 3T3 fibroblast cell line, may not represent the behavior of all fibroblast types. Notably, as mentioned before, we previously found that human bronchial fibroblasts, which exhibit electrotaxis towards the anode, contrasting with the cathodal migration observed in 3T3 cells, rely on the TGF-β signaling pathways (Pavlenko et al., 2023).

While we focused on TGF-β and PI3K pathways, alternative signaling mechanisms such as ion channels and other growth factor receptors require further exploration. Our recent publication on ionic mechanisms and EGFR involvement in 3T3 electrotaxis has begun to explore these additional pathways (Lasota et al., 2024). Further investigations will focus on the dynamics of membrane receptors redistribution, aiming to validate the biphasic model proposed in our work.

Although specific *in vivo* experiments concerning the detailed cellular mechanisms of electrotaxis may be challenging, the application of electrostimulation in treating hard-to-heal wounds underscores the clinical relevance of understanding these mechanisms (Martin-Granados and McCaig, 2013). A deeper comprehension of electrotaxis could significantly enhance the efficacy of combining electrostimulation with pharmacological interventions in wound care. This integrated approach could lead to more targeted and effective treatments, paving the way for innovative therapeutic strategies in wound management.

## Conclusion

Our study has shown that inhibition of both canonical and several non-canonical signaling pathways associated with the activated TGF-β receptor did not impede the directional migration of 3T3 cells to the cathode. Furthermore, silencing the expression of the TGF-β receptor failed to eliminate the directional migration of 3T3 cells in the electric field. Additionally, there was no redistribution of the TGFβ receptor under electric field conditions. Nonetheless, our research confirms an important role for PI3K kinase in electrotaxis, but its activation in our model was probably related to the action of factors other than TGFβ.

## Data Availability

The raw data supporting the conclusion of this article will be made available by the authors, without undue reservation.
