# Supplementary figures and images for: The role of TGF-β in the electrotactic reaction of mouse 3T3 fibroblasts in vitro
Source: Acta Biochim Pol. 2024 Jun 25;71:12993. doi: 10.3389/abp.2024.12993 (PMC11231101; doi:10.3389/abp.2024.12993)

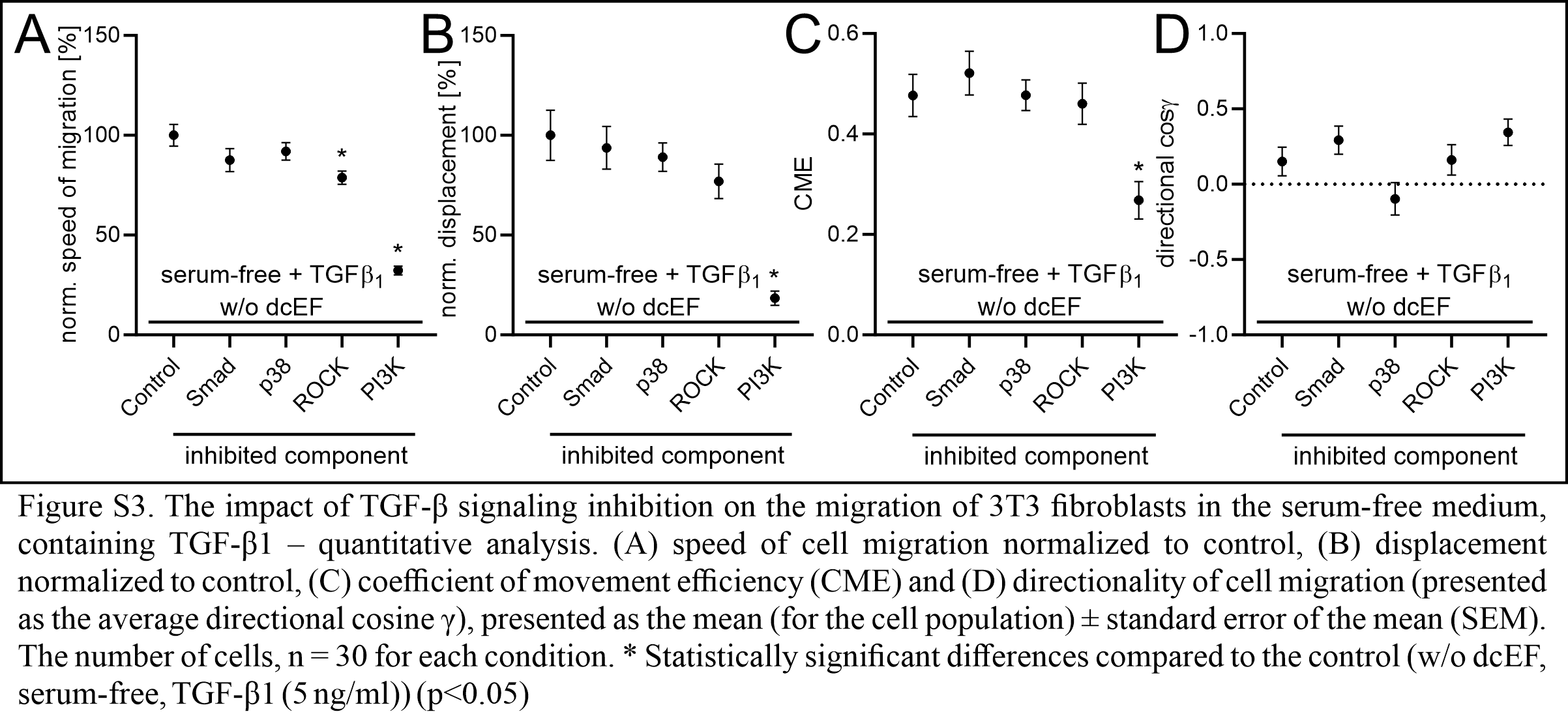

Supplement: Supplementary file 1 [file Image3.TIF]

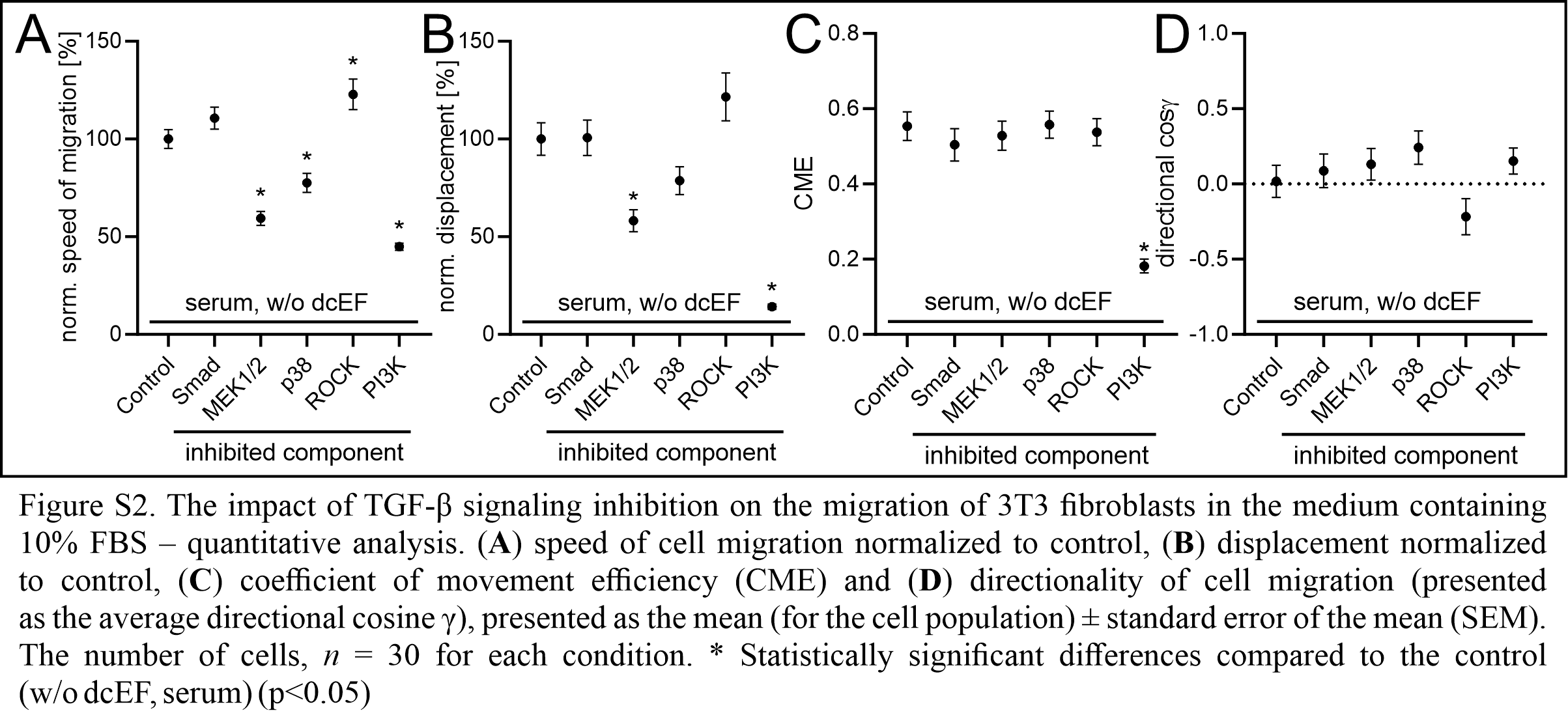

Supplement: Supplementary file 2 [file Image2.TIF]

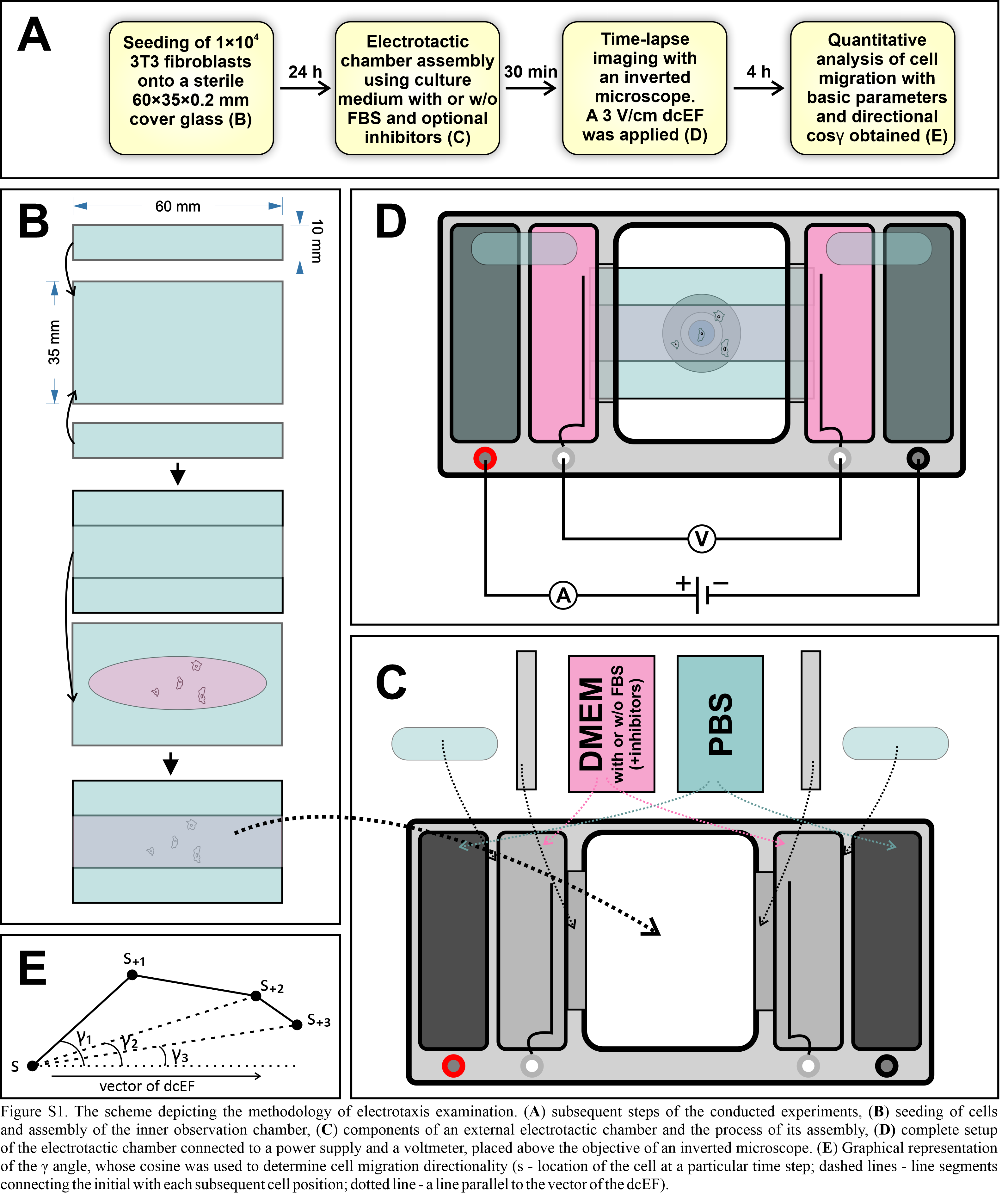

Supplement: Supplementary file 3 [file Image1.TIF]
